# Integrative cross-species analysis of GABAergic neuron cell types and their functions in Alzheimer’s disease

**DOI:** 10.1038/s41598-022-21496-7

**Published:** 2022-11-11

**Authors:** Shiyou Wang, Peiwen Ding, Jingnan Yuan, Haoyu Wang, Xiuqing Zhang, Dongsheng Chen, Dongli Ma, Xingliang Zhang, Fei Wang

**Affiliations:** 1 College of Life Sciences, University of Chinese Academy of Sciences, Beijing, 100049 China; 2 BGI-Shenzhen, Shenzhen, 518083 China; 3 Department of Respiratory Diseases, Institute of Pediatrics, Shenzhen Children’s Hospital, Shenzhen, 518038 China; 4 Department of Pediatrics, The Affiliated Hospital of Guangdong Medical University, Zhanjiang, 524001 China; 5 Department of Biomedicine, Aarhus University, Aarhus, Denmark

**Keywords:** Cell biology, Evolution, Neuroscience, Systems biology

## Abstract

Understanding the phenotypic and functional diversity of cerebral cortical GABAergic neurons requires a comprehensive analysis of key transcriptional signatures and neuronal subtype identity. However, the diversity and conservation of GABAergic neurons across multiple mammals remain unclear. Here, we collected the single-nucleus RNA sequencing (snRNA-seq) datasets of cerebral cortex from human, macaque, mouse, and pig to identify the conserved neuronal cell types across species. After systematic analysis of the heterogeneity of GABAergic neurons, we defined four major conserved GABAergic neuron subclasses (Inc *SST*, Inc *LAMP5*, Inc *PVALB*, and Inc *VIP*) across species. We characterized the species-enriched subclasses of GABAergic neurons from four mammals, such as Inc Meis2 in mouse. Then, we depicted the genetic regulatory network (GRNs) of GABAergic neuron subclasses, which showed the conserved and species-specific GRNs for GABAergic neuron cell types. Finally, we investigated the GABAergic neuron subclass-specific expression modules of Alzheimer’s disease (AD)-related genes in GABAergic neuron cell types. Overall, our study reveals the conserved and divergent GABAergic neuron subclasses and GRNs across multiple species and unravels the gene expression modules of AD-risk genes in GABAergic neuron subclasses, facilitating the GABAergic neurons research and clinical treatment.

## Introduction

Neurons in the mammalian central nervous system (CNS) are numerous and diverse, contributing critically to the stable maintenance of neuronal networks. GABAergic inhibition is an important regulator of excitability in neuronal network activity^1^. GABAergic neurons produce gamma-aminobutyric acid (GABA), acting as the main inhibitory neurotransmitter in the CNS^2–4^. The molecular and functional properties and heterogeneity of GABAergic neurons have been characterized by synaptic communication diversification^5^. However, there is still a lack of comprehensive understanding of the similarities and divergences for GABAergic neuron cell types, especially the GABAergic neuron cell heterogeneity across species.

Unlike conventional sequencing methods, single-cell/nucleus RNA sequencing (scRNA-seq/snRNA-seq) technologies can accurately profile transcriptomic characterization of individual whole cell and nucleus, respectively. Especially, both scRNA-seq and snRNA-seq enable profiling the complete cellular makeup in the brain, classifying the cell type heterogeneity of neurons^6–9^. The GABAergic neuron cell types in the cerebral cortex for human^10,11^, macaque^12,13^, mouse^14,15^, and pig^16^ were distinguished at the single-cell level. Moreover, the conserved transcriptional programs for the development of GABAergic neurons were recently characterized in mouse and human^17^. Another study showed the orthologous cell types of GABAergic neuron subtypes and the conserved molecular features of GABAergic chandelier neurons across human, marmoset, and mouse^18^. While continuous progress is emerging in understanding the development and evolution of GABAergic neurons, the studies elucidating the conservation and functional diversity of GABAergic neurons across multiple mammalian species are still valuable for GABAergic research in animal kingdoms.

Additionally, scRNA-seq data, along with computational analyses, can be innovatively used to investigate genetic regulatory networks (GRNs)^19^. GRNs underlie and maintain cell-type-specific transcriptional states, which provides the opportunities to understand the cell-type heterogeneity and functional features^20^. The differentiation, fate, and diversification of GABAergic neurons are controlled by the specific transcriptional architecture of synaptic communication^21^. The distinct transcription regulatory modules mainly specialized in specific GABAergic neurons have been characterized in mouse and human^22^. However, the conservation and divergences of GRNs in GABAergic neurons across multiple species are still unclear.

Genetic variation renders susceptibility to neurodevelopmental disorders (NDDs) by influencing the development of specific cell types. Alterations in cortical GABAergic neurons are common in neuropsychiatric disease. Alzheimer’s disease (AD) is the increasingly common form of dementia in the aging population, characterized by memory decline in the initial stages and subsequent progressive deficits in cognition and behavior. Unfortunately, there are no attractive therapies interfering development and progression of AD because of many risk alleles in different neuron cell types. Knowledge of risk genes by genome-wide association studies (GWAS) is invaluable in understanding the complicated pathogenesis of AD^23^. The scRNA-seq technology can analyze cellular heterogeneity and identify gene expression profiles of different cell types. Analysis of the single-cell gene expression profiles in human AD showed that neurofibrillary tangles (NFTs)-bearing neuronal subtypes expressed a core set of 63 genes associated with synaptic vesicle cycle and transsynaptic signaling^24^. Based on the scRNA-seq data of human, macaque, mouse, and pig brains, elucidating the cortical neuronal-cell type-specific expression patterns of AD risk genes helps us understand the cognitive abilities and susceptibility to AD.

Here, we integrated snRNA-seq datasets to elucidate the heterogeneity of GABAergic neurons and comparatively analyze the GABAergic neuron subtypes across human, macaque, mouse, and pig. The regulatory network analysis was performed in GABAergic neurons across species, and the key regulatory modules were identified. We also systematically analyzed the expression patterns of AD-related genes in GABAergic neuron cell types.

## Results

### The heterogeneity of GABAergic neurons in human, macaque, mouse, and pig

**Figure 1:**
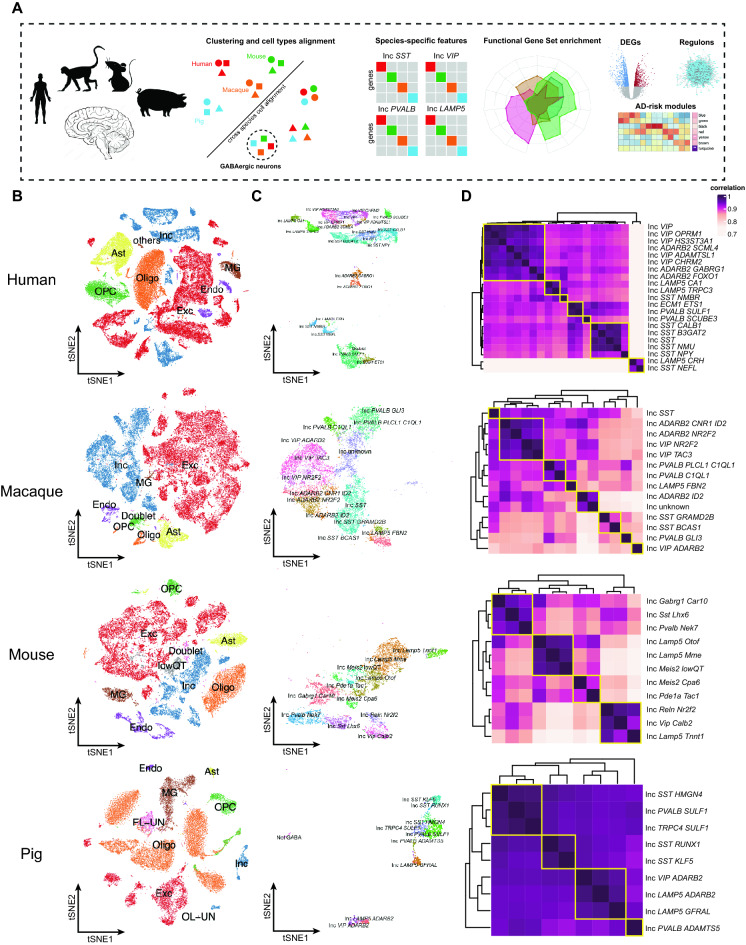
Cell type heterogeneity across species. (**A**) Pipeline of article design and data analysis. (**B**) The t-SNE plots show the major cell types in human, macaque, mouse, and pig. Exc, Excitatory neuron; Inc, Inhibitory neuron; MG, Microglia; Endo, Endothelial cells; OPC, Oligodendrocyte progenitor cells; Oligo, Oligodendrocytes; Ast, Astrocytes. (**C**) The t-SNE plots show the subtypes of GABAergic neurons in human, macaque, mouse, and pig. (**D**) The heatmaps show the correlation of different GABAergic neurons subtypes in human, macaque, mouse, and pig.

To perform a cross-species comparative study of the GABAergic neurons, we collected the snRNA-seq datasets of the cerebral cortex for human^10,11^, macaque^12,13^, mouse^14,15^, and pig^16^. After cell-type annotation and filtering out the excitatory neurons and non-neurons, the GABAergic neurons were used for heterogeneity, functional properties, and AD-related risk genes analysis across species (Fig. 1A, Table S1). Specifically, datasets from the same species were integrated using reciprocal principal component analysis (RPCA). After determining anchors between any two datasets using RPCA, we projected each dataset into the other PCA space and constrained the anchors by the same mutual neighborhood requirement. The datasets of the cerebral cortex for each species were integrated and visualized by t-SNE. The major cell types of oligodendrocyte progenitor cells, astrocytes, oligodendrocytes, endothelial cells, excitatory neurons, microglia, and inhibitory neurons were identified by the expression of canonical gene markers, suggesting the conservation of major cellular compositions across these four species (Figs. 1B, S1A).

Subsequently, the GABAergic neurons were split from each species for cell-type heterogeneity analysis. The GABAergic neuron subclasses were annotated by differentially expressed genes (DEGs). Four major subclasses of GABAergic neurons were identified with significant DEGs of *PVALB*, *SST*, *LAMP5,* and *VIP* for each subclass. These results are consistent with previous reports^22^. However, some species-specific GABAergic neuron subclasses were defined, such as human Inc *ECM1*, mouse Inc Meis2, Inc Pde1a, Inc Reln, and pig Inc *TRPC4*. We determined the distinct cell classes for these four GABAergic neuron subclasses according to the DEGs in each subclass. For example, human Inc *VIP OPRM1*, Inc *LAMP5 CA1*, Inc *PVALB SULF1*, Inc *SST NEFL* highly expressed specific *OPRM1*, *CA1*, *SULF1*, and *NEFL* independently (Figs. 1C, S1B). We then split the GABAergic neuron subclasses to calculate the average gene expression of each cell class for four different species. The results showed that the cell types were linearly proportional to the average gene expression, which confirmed that the subclasses of GABAergic neurons are highly consistent with average gene expression patterns in each cell type (Fig. 1D).

### Comparative analysis of GABAergic neuron subclasses across species

**Figure 2:**
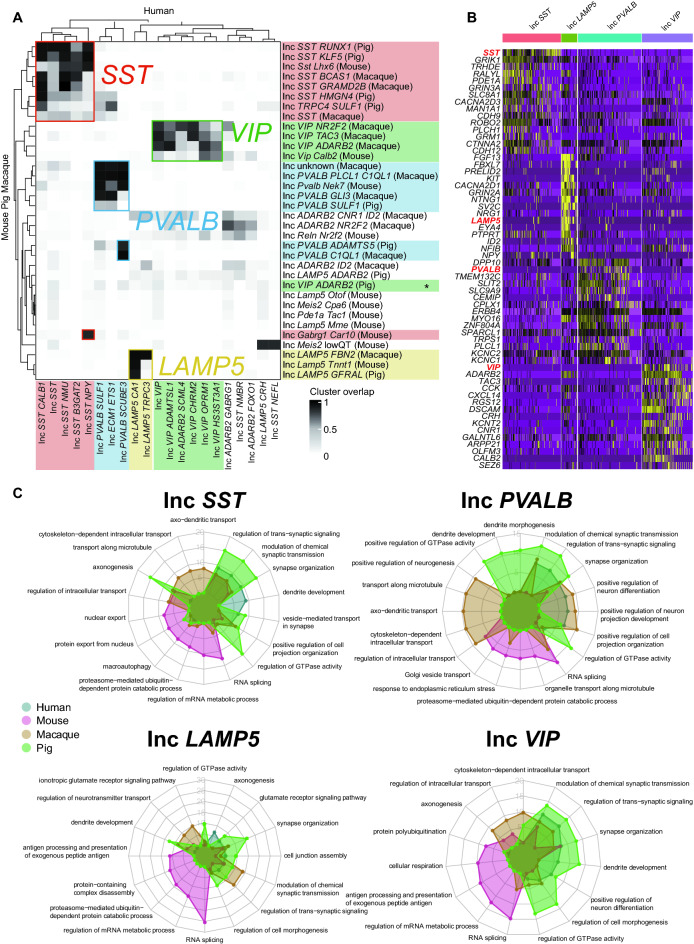
The conserved and diverse GABAergic subclasses across species and their functional features. (**A**) Heatmap showing the cluster overlaps of GABAergic subclasses between human and the other three species. (**B**) Heatmap showing top 15 DEGs of conserved GABAergic neuron types containing Inc *SST*, Inc *LAMP5*, Inc *PVALB*, and Inc *VIP*. (**C**) The GO terms are enriched from conserved and specific genes in the four species. Blue: Human; Red: Mouse; Brown: Macaque; Green: Pig.

To analyze the GABAergic neurons between different mammalian species, we calculated the homologous genes of macaque, mouse, and pig according to humans (Table S2). The subclasses of GABAergic neurons were annotated by the shared homologous genes and integrated between non-human species (macaque, mouse, and pig) and humans. The results showed that more GABAergic subclasses were identified in human than in non-human species (Fig. S2A). We further analyzed the conservation and divergences of four subclasses of GABAergic neurons across these four species. In the Inc *SST* subclass, the identified subtypes in non-human species were highly conserved with human, while two subclasses of Inc *SST* *NMBR* and *NEFL* from human were discrete. However, Inc *SST* subtypes within each non-human species were various overlapped with human. For example, Inc *SST RUNX1* and *KLF5* from pig were more conserved than Inc *SST HMGN4* compared to human Inc *SST*. Most of the Inc *VIP* subclass from macaque and mouse were highly conserved to the human, but the pig Inc *VIP* subclass was lowly conserved to human. The Inc *PVALB* subclasses from macaque and mouse were highly conserved with human, while pig Inc *PVALB SULF1* was lowly conserved to human. For the Inc *LAMP5* subclass, all the macaque, mouse, and pig subtypes were highly conserved to the human Inc *LAMP5 CA1* subtype, and only macaque Inc *LAMP5 FBN2* was conserved to the human Inc *LAMP5 TPPC3* subtype. Interestingly, mouse-specific Inc Meis2 were highly conserved to human Inc *LAMP5 CRH* and Inc *SST NEFL* (Fig. 2A). To confirm the conservation of GABAergic neuron subclasses across four mammalian species, we analyzed the split inhibitory neuron cells by their DEGs. The results demonstrated that *SST*, *LAMP5*, *PVALB,* and *VIP* were highly expressed in the top 15 gene list, confirming the four major GABAergic neuron subclasses of Inc *SST*, Inc *LAMP5*, Inc *PVALB*, and Inc *VIP* across species (Figs. 2B, S2B). While the four major subclasses of GABAergic neurons were notably conserved across species, the differences in GABAergic neuron cell subclasses between different species were also depicted, which could be demonstrated by differential gene expression in the subclasses.

Changes in gene expression patterns drive individual phenotypic differences and the evolution of new phenotypes between species^25,26^. To investigate cross-species functional conservation and divergences for GABAergic neurons, we performed GO (Gene Ontology) term enrichment analysis of four GABAergic neuron subclasses in human, macaque, mouse, and pig. While most terms across species showed highly conserved in four species, in Inc *SST* subclass, the cell types from pig are more divergent than the other three species, especially for the functions of axonogenesis, regulation of GTPase activity. However, some of functional terms are conserved in different species, such as regulation of intracellular transport terms detecting in macaque and pig, regulation of trans-synaptic signaling, modulation of chemical synaptic transmission, synapse organization detecting in all four species. In Inc *PVALB* subclass, the functional terms from three non-human species showed more discrete than human. For example, the cell types from pig showed some species-specific functions of regulation of GTPase activity, dendrite development, dendrite morphogenesis, positive regulation of GTPase activity; macaque showed some specific functions of transport along the microtubule, axo-dendritic transport, positive regulation of neurogenesis, cytoskeleton-dependent intracellular transport; and mouse demonstrated species-specific functions of Golgi vesicle transport, proteasome-mediated ubiquitin-dependent protein catabolic process, and organelle transport along microtubule. In the Inc *LAMP5* subclass, the most divergences of cellular functional features from mouse to human are RNA splicing, regulation mRNA metabolic process, et al. In the Inc *VIP* subclass, human and pig showed more species-specific cell type functions than macaque. In addition, some functional terms are conserved in four species, such as modulation of chemical synaptic transmission, synapse organization. (Fig. 2C). These results showed the conservation of GABAergic neuron subclasses with functional diversity across human, macaque, mouse, and pig.

### Conservation and divergences of regulatory modules for GABAergic neurons across species

**Figure 3:**
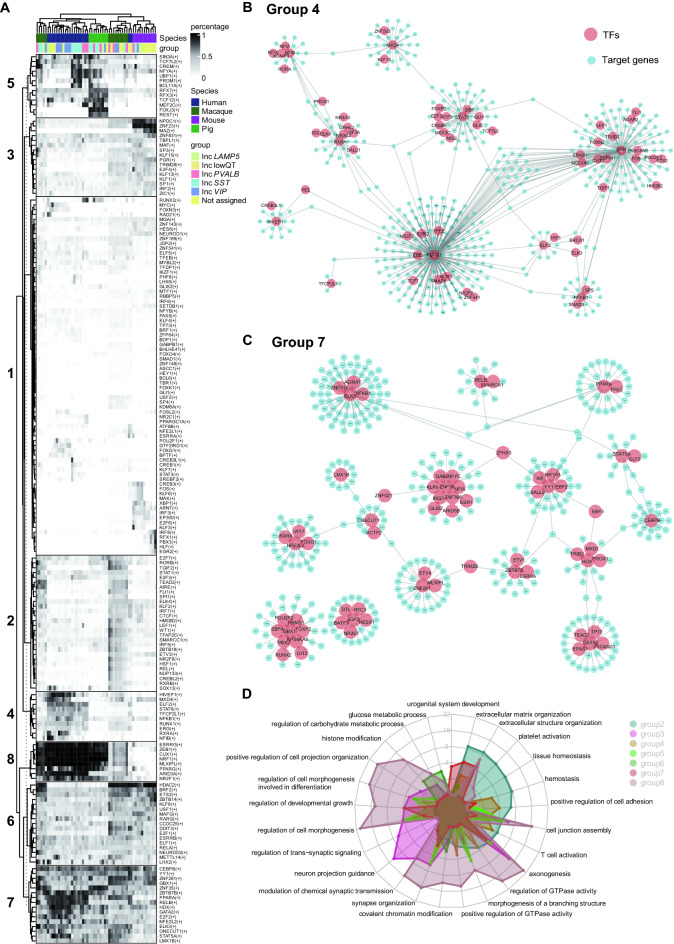
Conserved and specific regulons among four species. (**A**) Heatmap showing the percentage of regulon-activated cells in each subtype in different species. The top annotation indicated species and major cell types. Major regulons were clustered as 8 groups based on activated patterns. (**B**, **C**) TF-targets regulon networks in groups 4 and 7. Red: TFs; Blue: target genes. (**D**) GO terms enriched from genes of group2-8.

Comparative studies of gene expression levels and the evolution of gene regulation provide the compelling evidence that most gene regulatory patterns evolve under selective constraint^27^. Regulons, consisting of transcriptional factors (TFs) and cofactors regulating each other and their downstream target genes, are associated with characteristic functional transcriptional activities^28,29^. Based on the homologous genes among the four species, we identified 9277 homologous genes for constructing gene regulatory networks. Finally, 182 regulons activated in at least one cell which were used for downstream analysis. To find out distinct regulons in different GABAergic subtypes, we calculated the percentage of activated cells in each cluster. All regulons were clustered into eight groups with specific activated patterns, conserved in part of species or all four species. For example, the regulons in group 1 were not enriched in any species; the regulons in group 2 were conserved in macaque; the regulons in group 3 demonstrated in mouse and part of macaque; the regulons in group 4 were enriched in human; the regulons in group 5 partly conserved in human and pig. Interestingly, the regulons in groups 6 and 7 were highly conserved in all four species. However, the regulons in group 8 were conserved in most species, excluding mouse (Fig. 3A). These results demonstrated that the regulons exhibited the conserved and divergent features across species.

To identify the target genes of the regulons in each group, we performed the GRNs analysis for seven groups except non-conserved regulons in any species of the first group. The results showed that the conserved transcription factors (TFs) specifically regulated functional gene modules for each group, such as the regulons conserved in human regulating functional modules gene in group 4 and the conserved TF-target pairs across all four species in group 7. The conserved TF-targets were also investigated in other groups (Figs. 3B,C, S3A, Table S3. We further performed the functional enrichment analysis using the TF-target pairs enriched in different groups. The enriched terms in the identified groups showed the functional similarities and differences of TF-target pairs across species (Fig. 3D).

### GABAergic neuron cell-type-specific AD-associated risk gene module

**Figure 4:**
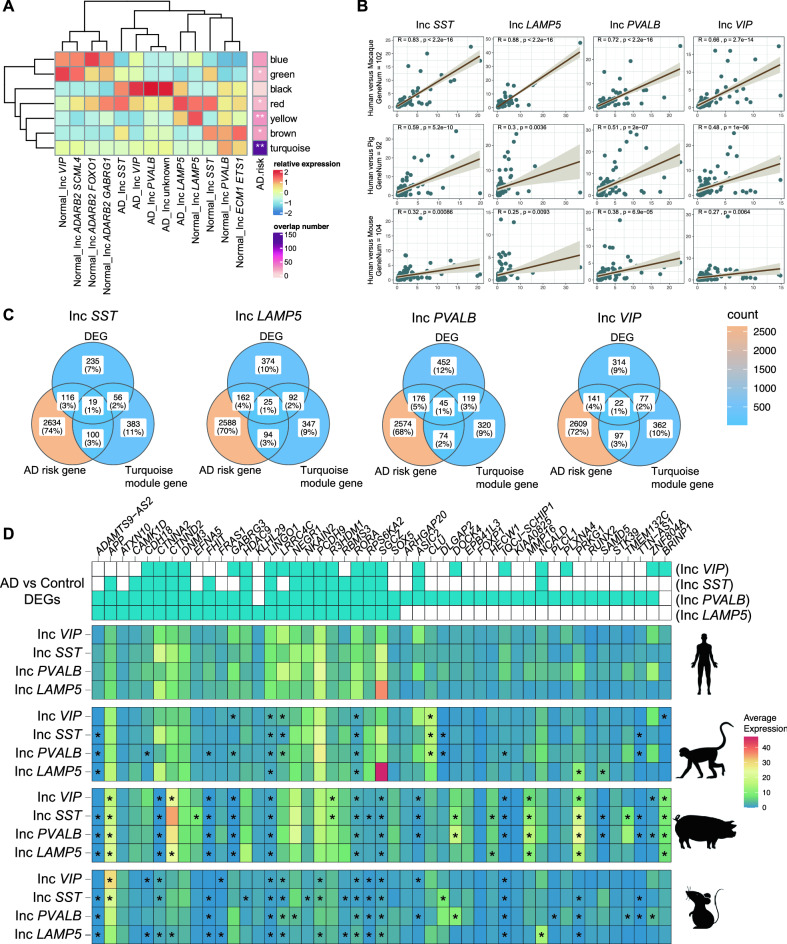
Critical AD-risk modules and GABAergic subtypes among four species. (**A**) Heatmap (left) showed relative expression of 7 WGCNA modules among different cell types. Each module expression was calculated as an average expression of all genes in the corresponding module and scaled by columns. Heatmap (right) showed the number of overlaps between module genes and AD high-risk genes, and the significance (*P* value) of the hypergeometric distribution test identified the module in which AD risk genes were over-represented (*P* value: 0.01–0.05, *; 0.001–0.01, **). (**B**) Spearman correlation of snRNA-seq expression of AD susceptibility genes showing the conservation of each cell type genes associated with AD between human and each of three species on four cell types. (**C**) Venn plots showing DEG overlaps in each cell type between AD and control, AD risk genes, and turquoise module genes. (**D**) The above heatmap displayed the DEGs in four GABAergic subtype neurons, in which blue represented significantly DEGs (adjusted *P* value < 0.05). Expression patterns of genes (columns) were associated with AD by cell type (rows) in four species. The degree of disease-associated gene enrichment in cell type was calculated by the Wilcoxon Rank Sum Test. *P < 0.05.

Genetic abnormalities in the central nervous system can lead to neurological disorders. AD is the most common neurodegenerative disease and the main cause of adult-onset symptoms of dementia. 2911 genes (Table S4) were associated with AD, which has been demonstrated by genome-wide association studies (GWAS) (https://www.ebi.ac.uk/gwas/)^30^. Analysis of gene co-expression modules by weighted gene co-expression network analysis (WGCNA) found that genes in the AD risk gene lists converged on seven modules. All modules exhibited noticeable changes in most cell types between normal and disease samples (Fig. 4A). Five of these seven modules were enriched for significant association with AD risk (*P* < 0.05, hypergeometric test). The module of particular interest intensively enriched for AD-associated risk genes was turquoise (Table S5), whose expression was evenly enriched in four inhibitory neuron subtypes (Inc *PVALB*, Inc *VIP*, Inc *LAMP5*, and Inc *SST*) irrespective of their origin (normal or disease samples). Next, we obtained the intersection between turquoise module genes and AD risk genes (119 genes, Fig. S4, Table S6), which were selected to understand the conservation of each cell type genes associated with AD. We compared the cell-type expression of these AD susceptibility genes between human and each of the other three species (Fig. 4B). Cell-type expression of the AD-associated genes in macaque displayed a high correlation with that in human. Except for the Inc *LAMP5*, the other three cell-type expressions of AD genes in pig showed moderate correlation with human AD susceptibility genes. Nevertheless, expression of AD-associated genes in four mouse cell types showed a very low correlation with those in human.

Finally, we obtained the intersection among turquoise module, AD risk genes, and DEGs (between control and AD samples). We found 19, 25, 45, and 22 AD-related disease genes in four inhibitory neuron cell types, respectively (Fig. 4C). We further assessed the cell-type expression of these genes in four species. We also found that the global expressional patterns of the four AD-related genes in four cell types were very similar between human and macaque (Fig. 4D). Many genes were conservatively expressed in four cell types of these two species but not in the other two species, such as *SGCZ* and *CTNNA2* (Fig. 4D), which was in good accordance with the abundant localization in all layers of the adult rhesus monkey dorsolateral prefrontal cortex by situ hybridization^31^. Intriguingly, a few genes such as *CLU* and *APP* in most cell types of macaque showed a higher expression level than those in human (Fig. 4D). Compared with mouse, pig showed more similar expression patterns to those in human (Fig. 4D).

## Discussion

In this study, we performed a comprehensive and comparative analysis of local single-cell transcriptome and regulatory modules of GABAergic neurons across multiple species in mammalian evolution. The GABAergic neurons with morphological, structural, and functional diversity are necessary for regulating the neural-circuit activity in the mammalian cerebral cortex. Single-cell transcriptomics analysis characterized the origins of diversity and molecular mechanism of development for GABAergic neurons in mouse and human ganglionic eminence^17,32^. Although the broad conservation of transcriptional programs of cardinal GABAergic neuron cell types and interneuron development were delineated in human versus mouse cortex^17,22^, the heterogeneity of GABAergic neurons across multiple mammalian cortexes is still unclear. Our study systematically analyzed the generally conserved cell types and species-enriched cell types of GABAergic neurons across human, macaque, mouse, and pig. Four conserved GABAergic neuron subclasses were identified: Inc *SST*, Inc *PVALB*, Inc *LAMP5*, and Inc *VIP*, indicating that the conserved cell-type architecture of GABAergic neurons widely exists in the mammalian cerebral cortex. While similar results were recently reported across human, marmoset, and mouse^18^, the interneurons in pig cerebral cortex compared to other mammals is still not well understood. The limited information on cell-type diversity and functional versatility for GABAergic neurons of cerebral cortex was reported previously^33^. Notably, the species-enriched GABAergic neurons subclasses, such as human Inc *ECM1*, mouse Inc Meis2 Inc Pde1a, Inc Reln, pig Inc *TRPC4,* were also identified. Among them, the Meis2-positive cells in mouse were found as a sub-population as lateral ganglionic eminence with progenitor cells features^34^. These results demonstrate the conserved and divergent transcriptional programs of GABAergic neurons across multiple mammals.

Genetic regulatory programs control embryonic development^35^ and the developmental process and functional diversity of cortical interneurons^2^. GABAergic neurons diversity and their functional maturation in cortical circuits are influenced by cell-intrinsic networks of transcription factors in progenitor cells, which is required for the development and specification of all GABAergic neurons in embryonic subpallium^36,37^. However, the characterizations of gene regulatory programs of GABAergic neurons across multiple mammals are still unclear. Our study systematically investigated the conserved and species-specific regulons across human, macaque, mouse, and pig. The identified transcription factors were clustered in eight groups with diverse regulatory modules across species. The homologous and conserved gene regulatory modules across all four species were identified, specifically into two groups with functional conservation. However, the distinct specific transcription factors in individuals or parts of four species were characterized. The evolutionary and conserved regulatory elements, including enhancers, promoters, and other regulatory elements across multiple species, were also analyzed and demonstrated that enhancers are rarely conserved across multiple mammalian species, but promoters showed more slow evolution^38^. Functional elements of GRNs also drive the phenotypic diversity and functional heterogeneity of subclass gene expression of neurons across species^37,39,40^. Thus, these results provided the fundamental information on homologous transcriptional factors that were conserved and divergent across four mammalian species, facilitating the understanding of the regulatory programs of GABAergic neurons in mammalian evolution.

Genetic mutations in the cerebral cortical neurons can cause neuropsychiatric diseases, as proved by many gene mutation experiments with a wide variety of phenotypes and GWAS^41,42^. However, it is difficult to accurately elucidate the molecular pathophysiological mechanisms at a certain cell type level by performing gene knockout at the whole level on animal models or in vitro cell function experiments. In contrast, scRNA-seq allows genome-wide profiling of the gene expressions of thousands of cells at one time, which has been previously performed in human^10,11^, macaque^12,13^, mouse^14,15^, and pig^16^ cortexes. Many genetic disorders account for AD dementia, the most common cognitive and functional degeneration with age^42^. It can be seen from Fig. 4 that the preferential expression of AD risk genes existed in a subclass set of cerebral cortical GABAergic neurons, highlighting the importance of choosing a reasonable animal for studying AD and other neuropsychiatric diseases in the future. Besides, treatment for neurological disorders is currently challenging and intractable. Recessive diseases represent the optimal candidates for gene therapy by replacing nonsense mutation^43^. For example, partial visual functional recovery was achieved in a patient blind due to retinitis pigmentosa after optogenetic gene therapy^44^. Cell transplantation or stem cell technology is promising to alleviate the progress of blinding diseases such as retinitis pigmentosa and age-related macular degeneration^45^. Therefore, our comparative analysis of multi-species cerebral cortical GABAergic neuron scRNA datasets provides the genetic context of AD susceptibility genes for each cell type individually.

In summary, the conservation and divergences of subclasses of GABAergic neurons were demonstrated with gene expression patterns in this study, specialized in the main cell types of Inc *SST*, Inc *PVALB*, Inc *LAMP5*, and Inc *VIP*. The conserved regulons, divided into eight groups, were identified across species, and their specific features in each group were characterized. Our study comprehensively deciphered cell-type-specific profiles of AD risk genes across four species, indicating future precise gene therapy for AD and other neurological disorders.

## Materials and methods

### Data collection

We collected snRNA-seq datasets of the cerebral cortex for comparative analysis of GABAergic neurons among four species: human, macaque, pig, and mouse. All datasets used in this study were downloaded from Gene Expression Omnibus (GEO), and the detailed information was described in Table S1.

### AD data collection

We collected AD data from the literature^24^ where researchers provided eight scRNA-Seq datasets, including three males and five females. To avoid the effect of gender, we selected three male datasets donated by Braak IV AD donors. In recent research, Vogel et al.^46^ divided AD patients into four subclasses based on distinct trajectories of tau deposition, including S1: limbic, S2: MTL-sparing, S3: posterior, and S4: lateral temporal. Our collected data was closer to S1: limbic, the limbic-predominant phenotype.

### Homogenous genes transformation from non-human species to human

Homogenous gene lists were downloaded from the database of Ensembl BioMart (https://grch37.ensembl.org/info/data/biomart/index.html). We used the “one to one” way to transfer macaque, mouse, or pig genes to human orthologues.

### MetaNeighbour analysis

To compare the ability of different gene families to distinguish cell types in mouse versus human, pig, or macaque brain cortex, we performed a modified supervised MetaNeighbour^47^ analysis by referring to a previous study^22^. We collected 442 HGNC gene sets, which their function might be associated with inhibitory neurons from Paul et al.^21^. We randomly selected 20 cells from each cluster to create a new object. Then, one dataset was divided into two groups by setting inhibitory neurons as 1 and others as 0. Besides, the batch effect was considered. Lastly, we used the function “run_MetaNeighbor” to calculate AUROC scores. After ten iterations, we obtained ten AUROC scores and determined the average AUROC and stander deviation as the performance of each gene set.

### Data integration by species

We collected several datasets of the brain cortex in each species in different batches. To identify the cell types in each species, we used the integrative method of reciprocal principal components analysis (RPCA)^48^ to remove batch effects in datasets and distinguish the same cell types from different datasets. In detail, all datasets from each species were merged into one dataset by the function “merge”. Then, the merged datasets were assigned to a raw data list with sample information. The data normalization was performed with a scale factor of 10,000. Variable genes were calculated based on the normalized data, and the top 3000 variable genes were chosen for downstream analysis. We used the function “FindIntegrationAnchors” to calculate the anchors of features for each merged dataset. To perform RPCA, we scaled and centered the 2000 features in each item, and 50 principal components (PCs) were obtained by PCA dimensionality reduction. When all steps were finished in each item, the function “FindIntegrationAnchors” was performed to get a set of anchors among these items with default parameters. After that, the dataset was projected into another PCA space and constrained the anchors by the same mutual neighborhood. Finally, the integrated object was calculated, and the integrated data was stored in the assay “integrated”. By setting the default assay as “integrated”, we scaled and centered the integrated data, and 30 PCs were obtained with a new PCA dimensionality reduction. All 30 PCs were put into dimension reduction for clustering and visualization by tSNE/UMAP.

### Cross-species cell type conservation estimation

We used the RPCA to identify the shared patterns among cells and aligned the similar cell types by gene expression cross-species. Firstly, the inhibitory neurons with the expression of *GAD1* and *GAD2* were extracted from the four species. With a combination of the differently expressed genes of subclusters and predefined cell types from Hodge et al.^22^, the inhibitory neurons were annotated into some cell subtypes. Then we randomly sampled 100 cells from each subtype (if the cell number of a subtype is less than 100, then use all of them). After gene symbol transformation from non-human species to human, the cell types from non-human species were integrated with that of human. The parameters for integration were the same as above, except only the top 2000 variable genes were selected in inhibitory neurons for analysis. The integrated data was then clustered into more than 30 clusters. To estimate overlap between cell type A of human and cell type B of mouse/pig/macaque, we first found out which clusters A and B were distributed and determined the shared clusters. The overlap was defined as the sum of the minimum proportion of samples in each cluster that overlapped within each RPCA cluster. Cluster overlaps ranged from 0 to 1 and were visualized in a heatmap with mouse/pig/macaque subtypes in a row and human subtypes in a column.

### Correlation analysis of inhibitory neuron subtypes

The correlation analysis between different inhibitory neuron subtypes was shown in Fig. 1D. The average gene expression of each GABAergic subclasses (R function: base: rowMeans()) was used for calculating the correlation between each pair of subtypes. The function of “cor” was performed with default parameters.

### GO term enrichment analysis

GO term enrichment analysis was performed using the packages “clusterProfiler”^49^ and “org.Hs.eg.db”. In detail, the gene symbol was transformed into Entrez id using the function “select”. The list of entrez id was used for GO term enrichment using the function “enrichGO” by setting the parameter as “BP” to find out all terms about biological process. We used the function “simplify” with default parameters to remove redundant terms with too many shared genes. The top 5 significant terms in each subtype were visualized.

### SCENIC analysis and visualization of regulons

We used pyscenic^50^ to find out regulons in different species. Four groups of inhibitory neurons were merged, and the shared homologous genes among four species were used for downstream analysis. Firstly, we used “GRNBoost2” to obtain the TFs and their target genes, defining the regulons. The regulons were generated using gene inference methods, which solely rely on gene expression correlations across cells. Secondly, the regulons were refound by pruning targets, which were absent the enrichment of a corresponding motif. TF effectively separation from d direct or indirect targets was based on cis-regulatory footprints. Finally, the original cells were assigned and clustered according to the activity of these discovered regulons. The regulon activities were binarized based on the bimodal model. After getting the matrix of different regulon activities, we calculated the proportion of activated cells in each subtype to create a new activity matrix. We used the K-means clustering method to cluster all regulons as eight groups based on the specific patterns. In detail, the K-means clustering method is a vector quantization method that minimizes within-cluster variances (squared Euclidean distances), but not regular Euclidean distances. We needed to determine the number of clusters before clustering. We artificially set the clustering number to 8. Visualization of the heatmap was finished using ComplexHeatmap^51^ with the parameters “row_km = 8”.

All regulons belonging to a group were visualized using cytoscape^52^. The points of TFs were colored red, while the other points were blue.

### WGCNA analysis and risk genes enrichment

We collected the AD scRNA-seq dataset from Garcia et al.^24^. In detail, eight AD samples were collected. Since the gender of normal human data was male, we selected the three male samples from these eight AD samples to minimize the effect of gender.

To identify potential gene modules associated with AD risk genes, we did the WGCNA^53^ analysis using the inhibitory neurons of AD and normal datasets in humans. Firstly, we merged two groups of datasets and calculated the top 2000 variable genes in each group. The intersection between the union of two sets of variable genes and the shared genes of two groups was finally used for downstream analysis. To minimize the effect of sparsity in a matrix of the scRNA-seq dataset, we merged ten cells into a pseudocell using the average expression in each subtype. Pseudocells of less than ten cells were excluded. According to the pseudocell matrix, the function “mad” was used to calculate the median absolute deviation of each gene and filter genes whose median absolute deviation was less than 0.1. The function “blockwiseModules” was used to gain potential modules by setting TOMType assigned, corType as pearson, power as 6, minModuleSize as 10, maxPOutliers as 1, and mergeCutHeight as 0.25.

The heatmap was plotted using the package “ComplexHeatmap”^51^. The color of the left heatmap was the column-scaled average expression of all module genes while the right heatmap color was the number of genes in each module.

The risk genes were collected from a website (https://www.ebi.ac.uk/gwas/)^30^. The *P* values of enrichment were calculated by 1-phyper (k − 1, M, N–M, n) with setting N as 20,000, M as the number of all AD risk genes, n as the number of genes in each module, and k as the number of intersections between genes in each module and AD risk genes.

### Correlation between human and other three species and visualization

We used the intersection of AD-risk genes and module genes to analyze the correlation between humans and the other three species. First, the average expression matrices in each cell type of used genes were calculated by using “AverageExpression” function from the Seurat package. Then, genes that were not homogenous from human were excluded. Finally, we used the “lm” methods to create fit curves and “Pearson” to calculate the correlations. The correlation curves between human and each of macaque, pig, and mouse were visualized by ggplot2^54^.

### Identification of the high confidence AD-risk genes across species

We used the “FindAllMarkers” to calculate the DEGs in each cell type between AD and healthy control human PFC. Then, we used the DEGs in each cell type to intersect the AD-risk genes and module genes as the high-confidence AD-risk genes. The R package “ggplot2”^54^ was used to visualize the expression of these genes. Moreover, the default method “Wilcoxon Rank Sum test” in the “FindMarkers” function was used to identify DEGs between human and macaque, mouse, and pig. Parameters of “min.pct” and logfc.threshold were set as 0.25 to filter infrequently expressed genes. The different expressions of high confidence AD-risk genes between human and each of macaque, pig, and mouse were signed in Figs. 4C, S4. The *P* values were calculated using “FindMarkers” and adjusted by using “BH” method. The adjusted *P* value less than 0.05 and the average value of logFC less than − 1 or more than 1 were labeled with *.

## Supplementary Information

Supplementary Information 1.

Supplementary Information 2.

Supplementary Information 3.

Supplementary Information 4.

Supplementary Information 5.

Supplementary Information 6.

Supplementary Information 7.

Supplementary Information 8.

Supplementary Information 9.

Supplementary Information 10.

Supplementary Information 11.

## Data Availability

All datasets in this study were obtained from published datasets. The information for datasets was supplied in Supplementary Table 1. We state that the data is available for purposes of peer review if requested by reviewers, within the terms of a data use agreement, and if compliant with ethical and legal requirements.

## Code availability

All codes used in this study are deposited on Github (https://github.com/Shiywa/GABAergic-neuron-conservation-cross-species/tree/main).
