# Supplementary figures and images for: Integrative cross-species analysis of GABAergic neuron cell types and their functions in Alzheimer’s disease
Source: Sci Rep. 2022 Nov 11;12:19358. doi: 10.1038/s41598-022-21496-7 (PMC9652313; doi:10.1038/s41598-022-21496-7)

# Figure S1

**A**

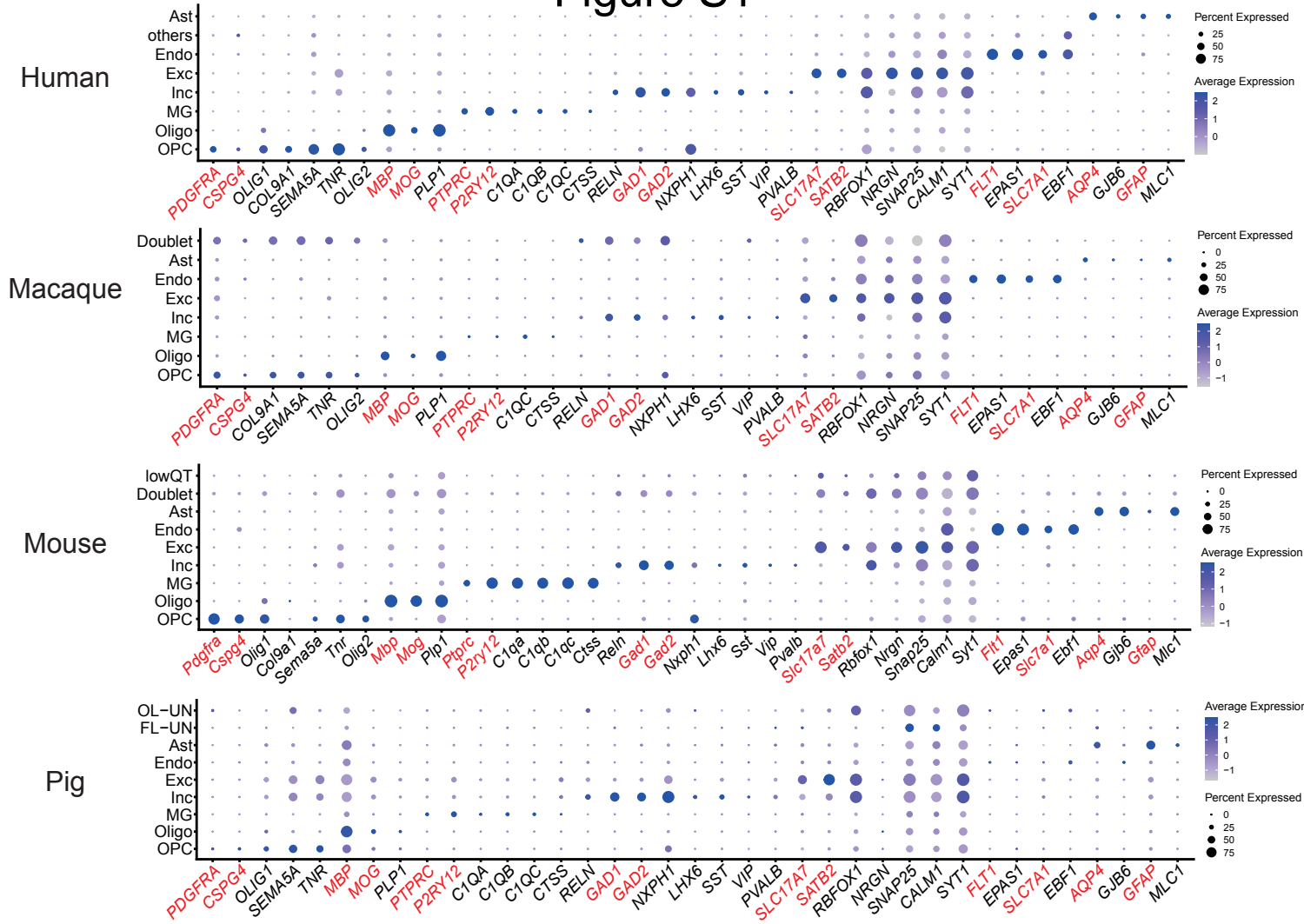

**B**

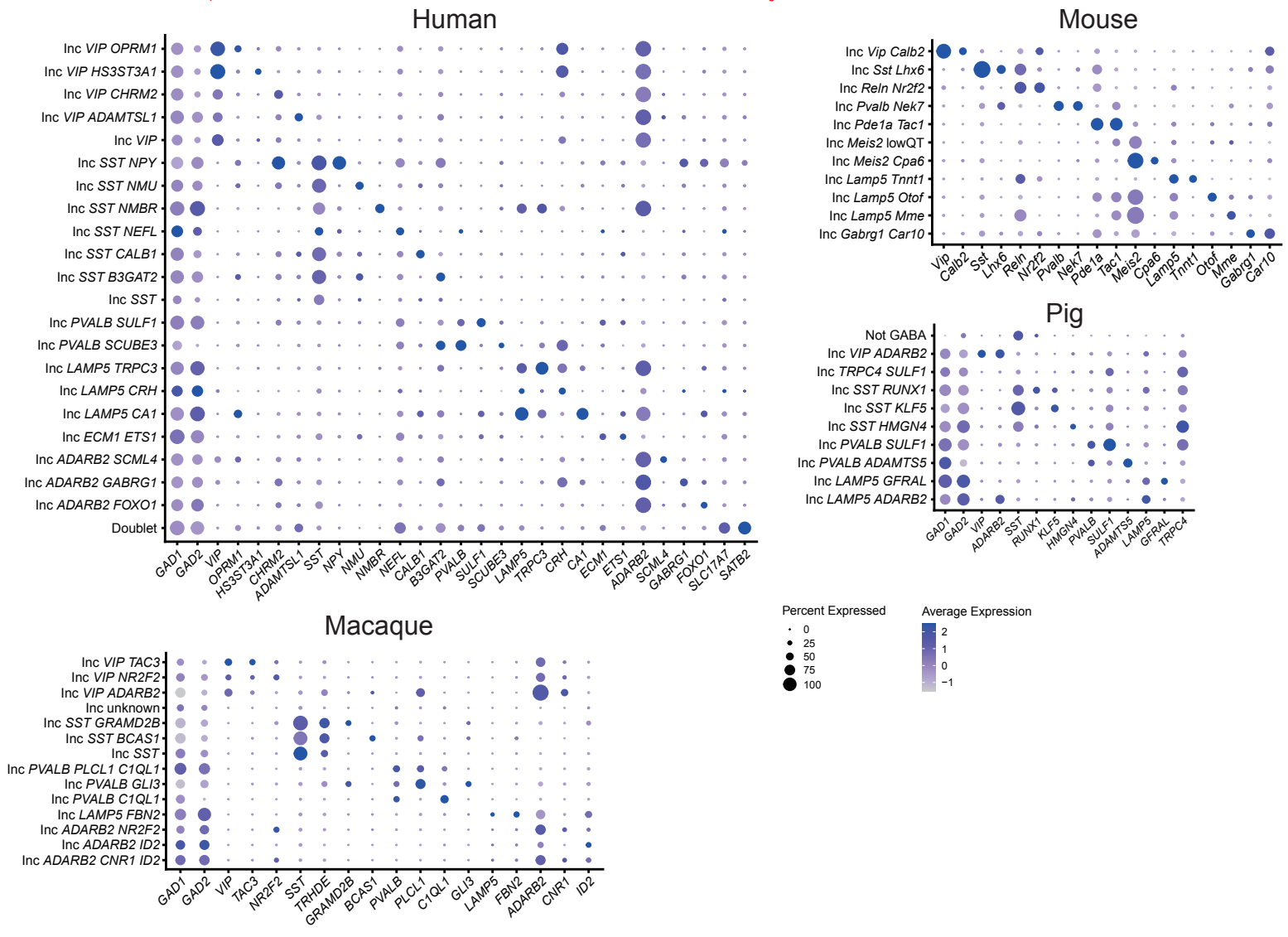

Supplement: Supplementary file 1 — Supplementary Information 1. [file 41598_2022_21496_MOESM1_ESM.pdf]

A

Figure S2

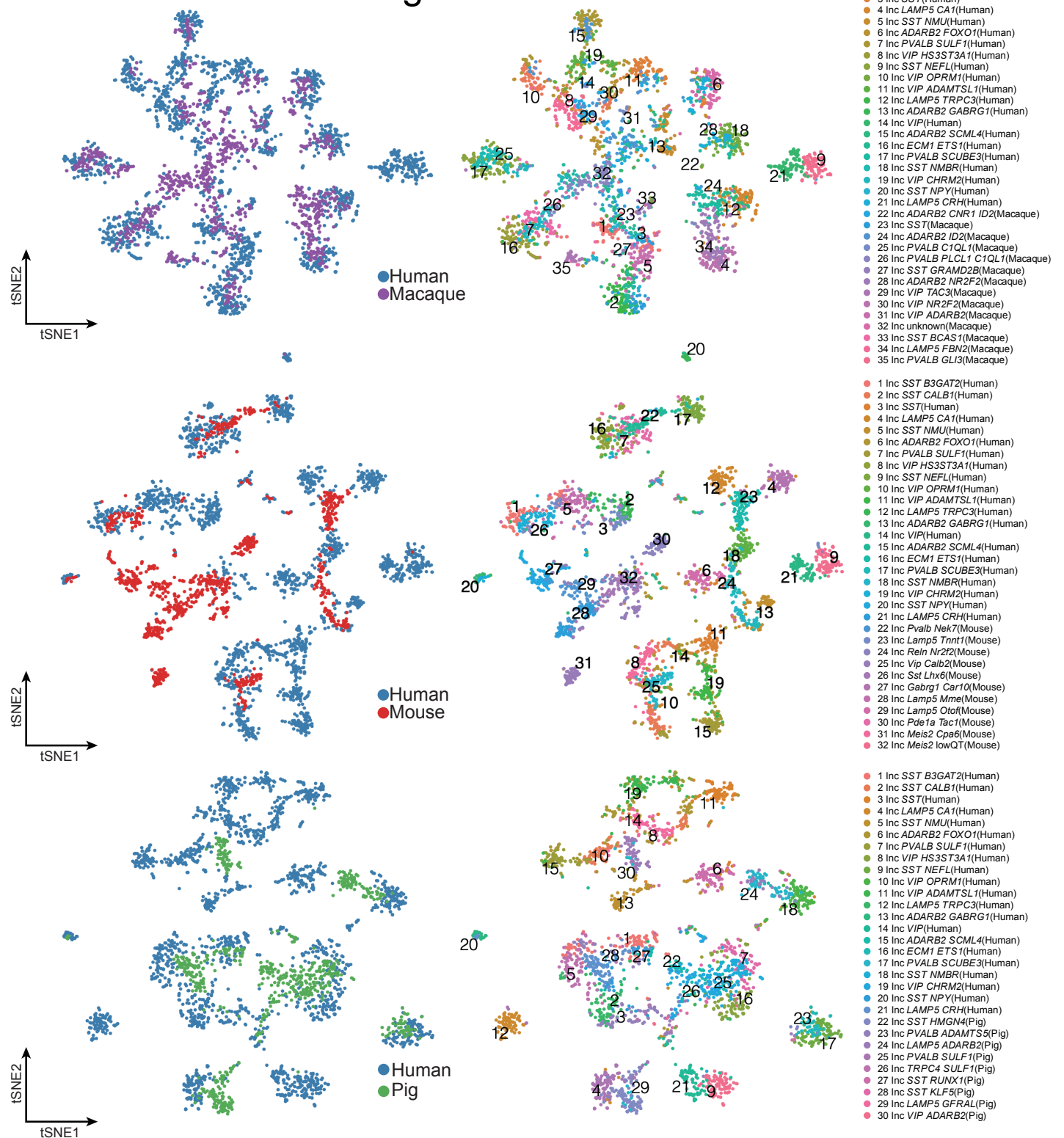

B

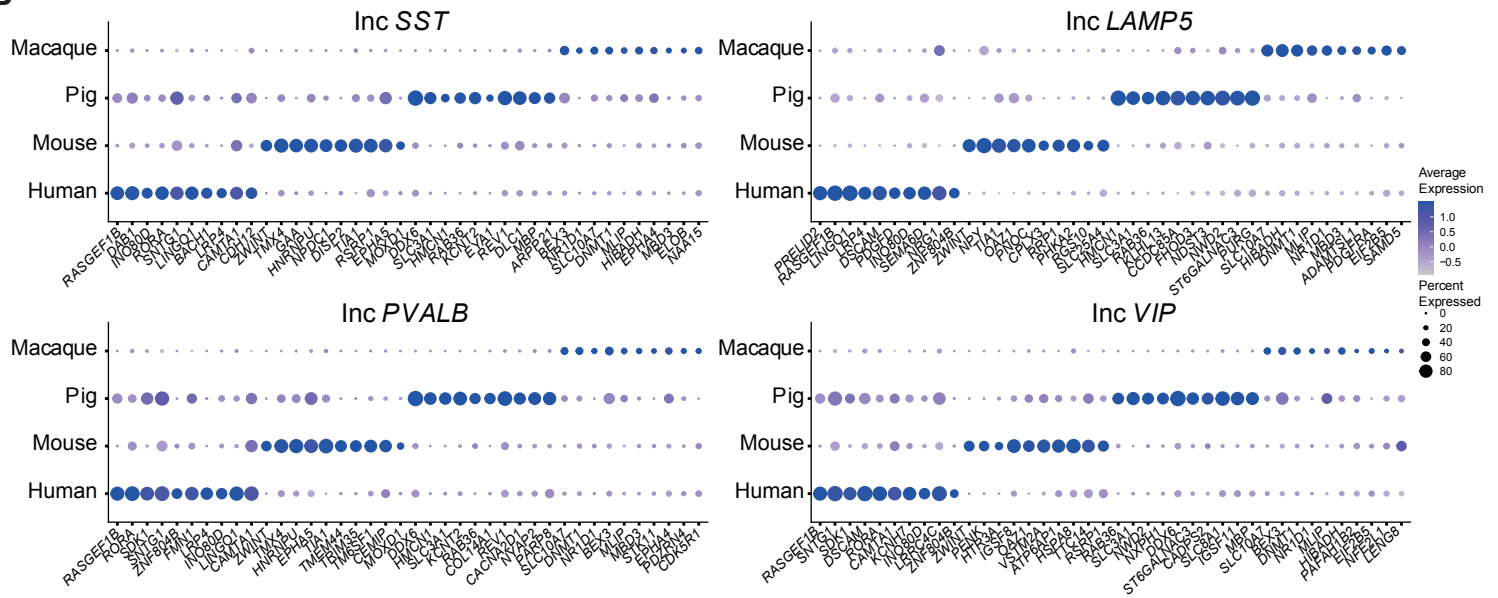

Supplement: Supplementary file 2 — Supplementary Information 2. [file 41598_2022_21496_MOESM2_ESM.pdf]

# Figure S4

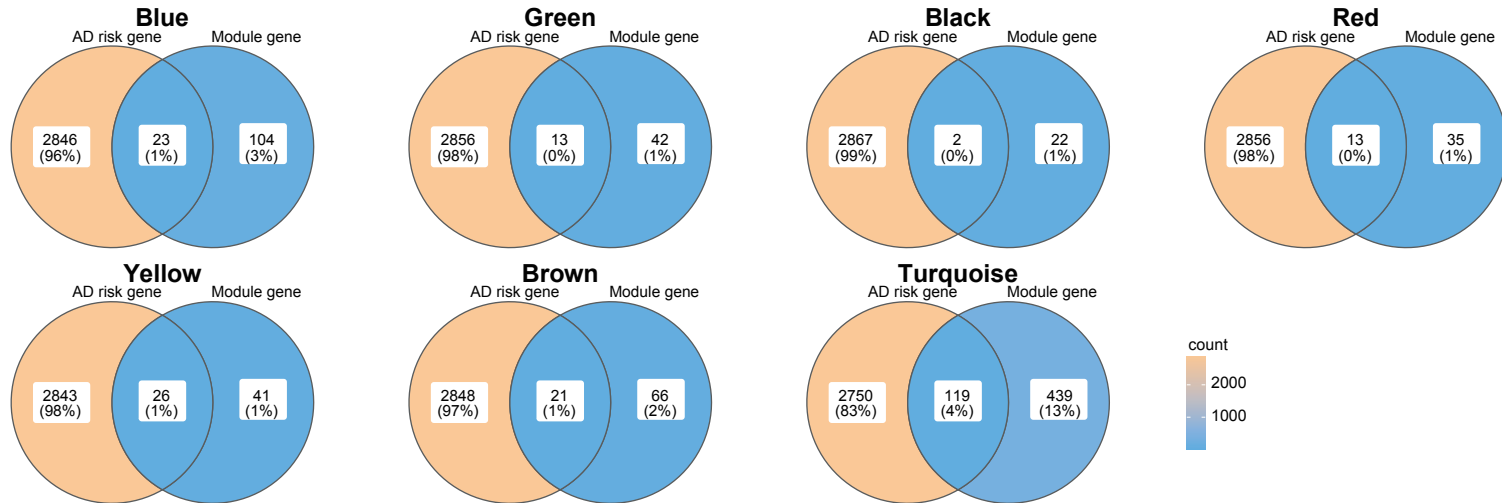

Supplement: Supplementary file 3 — Supplementary Information 3. [file 41598_2022_21496_MOESM3_ESM.pdf]

## Group2

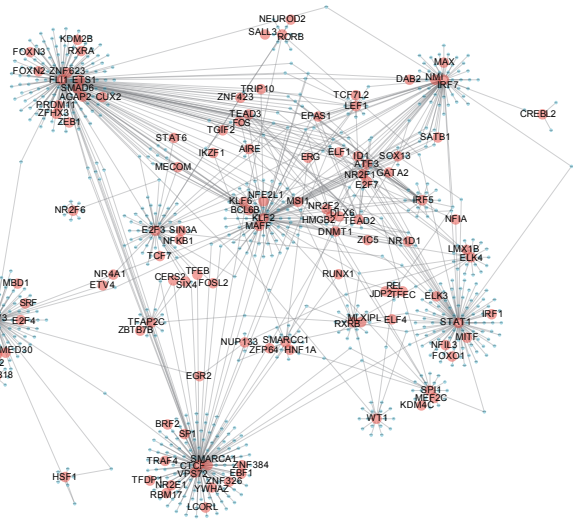

## Group 3

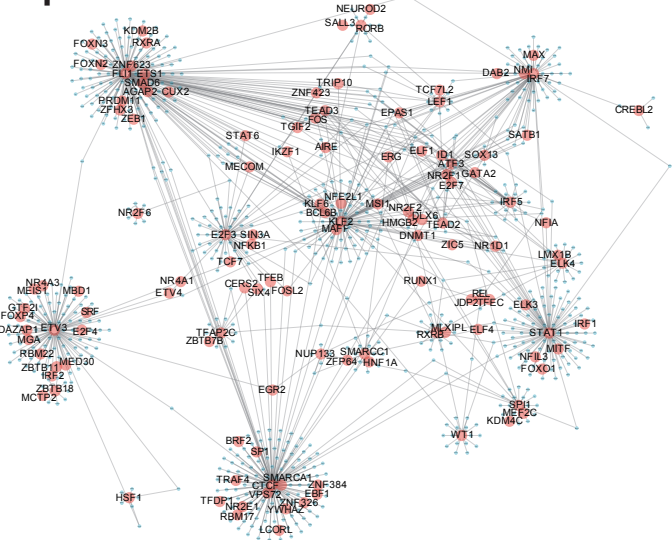

## Group 5

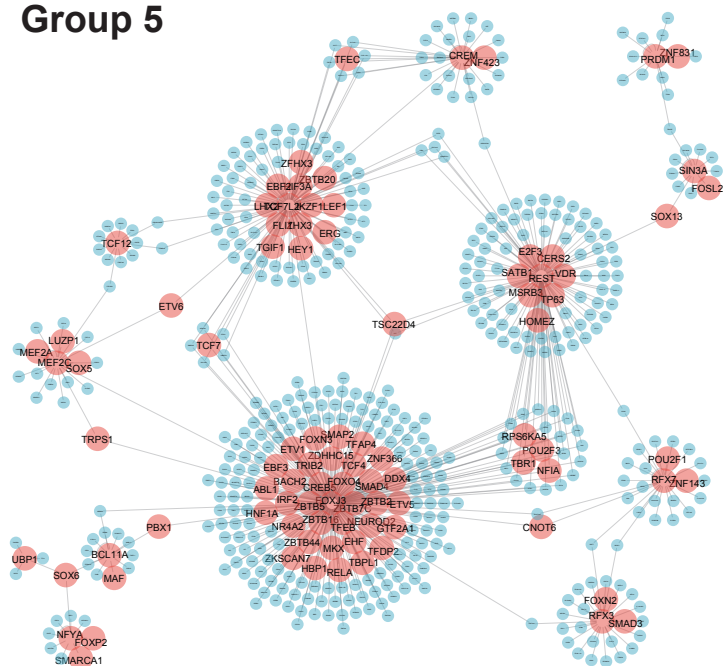

## Group 6

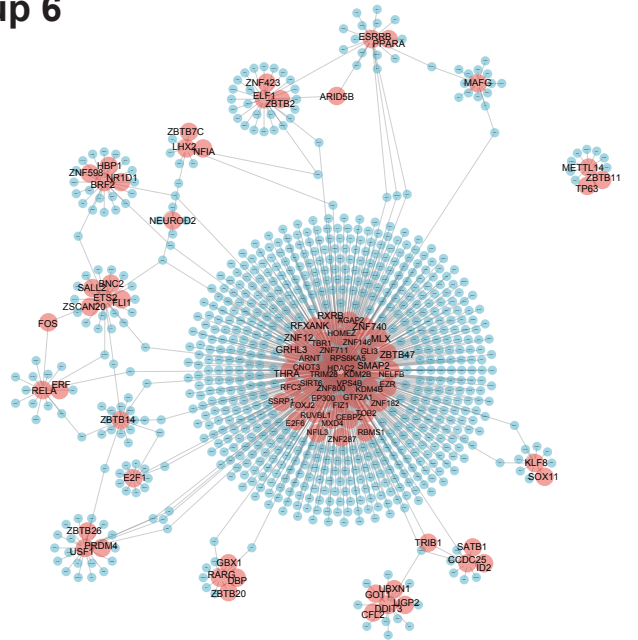

## Group 8

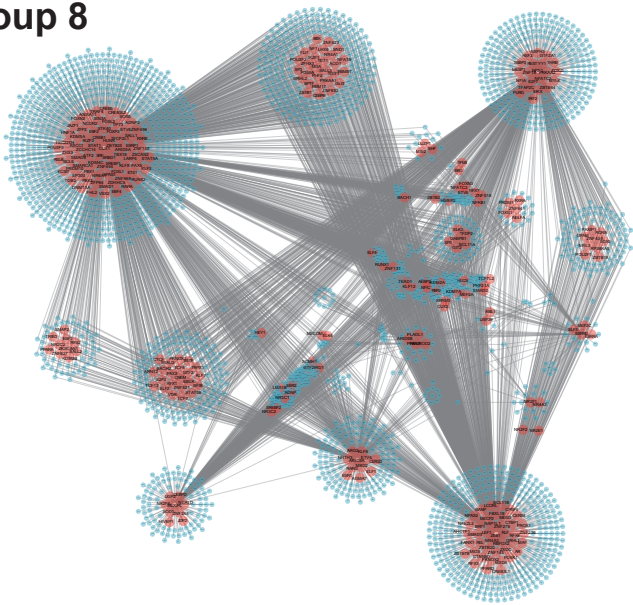

Supplement: Supplementary file 4 — Supplementary Information 4. [file 41598_2022_21496_MOESM4_ESM.pdf]
