# Supplementary material for: Integrative cross-species analysis of GABAergic neuron cell types and their functions in Alzheimer’s disease
Source: Sci Rep. 2022 Nov 11;12:19358. doi: 10.1038/s41598-022-21496-7 (PMC9652313; doi:10.1038/s41598-022-21496-7)
Supplement: Supplementary file 5 — Supplementary Information 5. [file 41598_2022_21496_MOESM5_ESM.docx]

**Supplementary Figure Legends**

**Fig. S1 Expression of cell type-specific markers.** A. Dot plots showing the expression of canonical markers of each cell type. B. Dot plots showing the expression of subtype-specific markers.

**Fig. S2 Comparative analysis of GABAergic neuron subclasses from non-human species to human**. A. t-SNE plots (left) showing the alignment between human and the other three species (macaque, mouse, and pig), respectively. t-SNE plots (right) show different types of GABAergic neurons. Each color represents one subtype. B. Dot plots showing the expression of top 10 species-specific genes in each cell type.

**Fig. S3** TF-targets regulon networks in groups 2, 3, 5, 6, and 8. TF-targets regulons were clustered into eight groups based on regulatory activity. Different groups had different activated patterns in four species.

**Fig. S4** Venn plots of the intersection of different module genes and AD-risk genes.

**Table S1** scRNA-seq/snRNA-seq datasets of cerebral cortex from human, macaque, mouse, and pig.

**Table S2** Homologous genes between non-human species and human.

**Table S3** TF-target pairs of GABAergic neuron subclasses across species.

**Table S4** The published AD-risk gene traits.

**Table S5** The intersection of module genes and AD-risk genes.

**Table S6** The intersect DEGs of module gene and AD-risk genes.
